# Clustering of Risk Factors for Non-Communicable Diseases among Adolescents from Southern Brazil

**DOI:** 10.1371/journal.pone.0159037

**Published:** 2016-07-19

**Authors:** Heloyse Elaine Gimenes Nunes, Eliane Cristina de Andrade Gonçalves, Jéssika Aparecida Jesus Vieira, Diego Augusto Santos Silva

**Affiliations:** Federal University of Santa Catarina, Research Center in Kinanthropometry and Human Performance, Florianópolis, Santa Catarina, Brasil; Indiana University, UNITED STATES

## Abstract

**Introduction:**

The aim of this study was to investigate the simultaneous presence of risk factors for non-communicable diseases and the association of these risk factors with demographic and economic factors among adolescents from southern Brazil.

**Methods:**

The study included 916 students (14–19 years old) enrolled in the 2014 school year at state schools in São José, Santa Catarina, Brazil. Risk factors related to lifestyle (i.e., physical inactivity, excessive alcohol consumption, smoking, sedentary behaviour and unhealthy diet), demographic variables (sex, age and skin colour) and economic variables (school shift and economic level) were assessed through a questionnaire. Simultaneous behaviours were assessed by the ratio between observed and expected prevalences of risk factors for non-communicable diseases. The clustering of risk factors was analysed by multinomial logistic regression. The clusters of risk factors that showed a higher prevalence were analysed by binary logistic regression.

**Results:**

The clustering of two, three, four, and five risk factors were found in 22.2%, 49.3%, 21.7% and 3.1% of adolescents, respectively. Subgroups that were more likely to have both behaviours of physical inactivity and unhealthy diet simultaneously were mostly composed of girls (OR = 3.03, 95% CI = 1.57–5.85) and those with lower socioeconomic status (OR = 1.83, 95% CI = 1.05–3.21); simultaneous physical inactivity, excessive alcohol consumption, sedentary behaviour and unhealthy diet were mainly observed among older adolescents (OR = 1.49, 95% CI = 1.05–2.12). Subgroups less likely to have both behaviours of sedentary behaviour and unhealthy diet were mostly composed of girls (OR = 0.58, 95% CI = 0.38–0.89); simultaneous physical inactivity, sedentary behaviour and unhealthy diet were mainly observed among older individuals (OR = 0.66, 95% CI = 0.49–0.87) and those of the night shift (OR = 0.59, 95% CI = 0.43–0.82).

**Conclusion:**

Adolescents had a high prevalence of simultaneous risk factors for NCDs. Demographic (gender and age) and economic (school shift) variables were associated with the most prevalent simultaneous behaviours among adolescents.

## Introduction

Non-communicable diseases (NCDs) have multiple causes (such as genetics and the environment), develop throughout life and have long-term characteristics. Estimates from the World Health Organization (WHO) indicate that NCDs account for more than half of global mortality, and in Brazil, these values reach 72.0% [1]. Thus, the United Nations (UN) has set a global commitment to face NCDs and prevent the factors that may increase the risk of developing these diseases [2]. During adolescence, mortality rates due to NCDs are extremely low; however, during this period, there is a concern about the impact that these diseases have in reducing school performance, social relationships, self-esteem and daily activities [3].

There is a high prevalence of hypertension (20.3%), abdominal obesity (30.0%), hyperglycaemia (14.4%) and dyslipidaemia (65.0%) found among Brazilian adolescents [4], and the risk of these diseases could be reduced by acting on modifiable risk factors for NCDs (such as physical inactivity, excessive alcohol consumption, smoking, sedentary behaviour and unhealthy diet). Behavioural factors are more sensitive to interventions than clinical factors [5]. Moreover, habits acquired at this stage of life tend to persist in adulthood [6–8].

An analysis of the distribution of health behaviours among adolescents (i.e., those 11–15 years old) from more than 100 countries found that approximately 80.0% of them performed daily physical activities (for at least 60 minutes), 32.0% used the computer for more than two hours per day, 6.0% smoked cigarettes daily, 7.6% consumed beer weekly and 25.0% had an unhealthy diet [9]. Each of these risk factors for NCDs has a different impact on health. Adolescents with low levels of physical activity have a higher risk of hypertension [3, 5]. Sedentary adolescents have a higher risk of obesity [10]. Physical inactivity and sedentary behaviour are different constructs and represent different causes and consequences that affect health [10]. Another risk factor for NCDs that has a negative impact on the health of adolescents is unhealthy diet, either through the low consumption of fruits and vegetables or the high intake of foods rich in sugars that cause diabetes mellitus type II [5, 7]. Additionally, excessive alcohol use causes changes in the central nervous system that may be a cause of depression among adolescents [9]. Smoking is responsible for respiratory diseases [7, 8].

Due to the influence of different demographic and economic realities on exposure to risk factors for NCDs [7], studies with young individuals have analysed the association between these variables [8,11]. Older adolescents [11], females [8], and students of the night shift [8] are more exposed to the simultaneous presence of risk factors for NCDs.

The investigation of the simultaneous presence of risk factors related to lifestyle is important because these risk factors are modifiable from changes in lifestyle. Few studies have analysed sedentary behaviour associated with other risk factors [12, 13], which are usually analysed separately in other studies. In addition, sedentary behaviour is highly prevalent among adolescents and identifying its relationship to other risk factors helps us understand and prevent the formation of habits at this stage of life [6, 14].

Assessing the association between clusters of risk factors and both demographic and economic characteristics is important to identify populations with a higher risk for the development of NCDs. Thus, the aim of the present study was to investigate the simultaneous presence of risk factors for NCDs (i.e., physical inactivity, excessive alcohol consumption, smoking, sedentary behaviour and unhealthy diet) and to verify their association with demographic and economic factors among adolescents of southern Brazil.

## Material and Methods

The population of this descriptive, cross-sectional and epidemiological study was composed of adolescents aged 14–19 years enrolled in public high schools of São José, Brazil. This study is part of a macro project entitled "Brazilian Guide for Evaluation of Physical Fitness Related to Health and Life Habits" [15, 16] and was approved by the Ethics Committee on Human Research of the Federal University of Santa Catarina (UFSC) under CAAE protocol: 33210414.3 .0000.0121. All procedures performed in the study were in accordance with the ethical standards of the national research committee, the 1964 Helsinki declaration and its later amendments as well as comparable ethical standards. Informed consent was obtained from all individuals included in the study and their guardians. The consent given on behalf of the enrolled children was written.

The city of São José is located in the Brazilian state of Santa Catarina in southern Brazil, and has a land area of 114.94 km^2^. In 2010, the city had a Human Development Index of 0.809, a percentage of young people (15–17 years old) with a complete primary education of 70.94%, an expected years of schooling of 10.52, a life expectancy at birth of 77.81 years, a per capita income of R$ 1.157,43, a Gini index of 0.44, a percentage of poor people of 1.36% and an infant mortality rate of 10% [17].

The sampling process was completed in two stages: stratified by public high schools (according to density) and clustered by classes considering school shift and grade. To determine the sample size, we followed the procedures suggested by Luiz and Magnanini [18] for a finite population. According to data from the Secretariat of Education of the State of Santa Catarina, we obtained 5,182 students (14–19 years old) who were enrolled in the 2014 school year at 11 eligible public schools of São José and distributed into 170 high school classes (74.8% of students were on the day shift). A confidence level of 1.96 (95% confidence interval), a tolerable error of five percentage points, a prevalence of 50%, and a 1.5 design effect [18] were adopted. We included an additional 20% to compensate for possible losses and refusals and another 20% to control for potential confounders in the association analyses [19]. Under these parameters, the required sample size was 751 students. However, as all students in the selected classes were included, this process resulted in sample larger than estimated, resulting in the collection of data from 1,132 students. For the present study, only individuals who answered all questions regarding the dependent and independent variables were considered, resulting in a sample of 916 adolescents.

The sample power for this study was calculated, showing values of 81.8% for the variable sex and 100.0% for the other variables (age, skin colour, school shift and economic level). Data collection was conducted in the second half of 2014.

### Dependent variable

The clustering of risk factors was identified from the evaluation of lifestyle behaviours: physical inactivity, excessive alcohol consumption, smoking, sedentary behaviour and unhealthy diet (Table 1). To classify individuals, a score ranging from 0 (no risk behaviour) to 5 (simultaneous presence of five risk behaviours) was used. Physical activity, excessive alcohol consumption and smoking behaviours were assessed by the Youth Risk Behaviour Survey (YRBS) questionnaire, which was translated and validated for Brazil [20]. Unhealthy diet was assessed by the Fantastic questionnaire (overconsumption of sugar, salt, animal fat, candies and snacks), which was translated, validated for Brazil and shown to have good reproducibility (0.92) and validity indicators (0.60 and 0.70) [21].

**Table 1:** Description and classification of dependent variables used in the study.

**VARIABLE**	**DEFINITION**	**QUESTIONNAIRE**	**CLASSIFICATION**
Physical inactivity	Not performing physical activity at least five times per week (60 minutes per day) last week.	YRBS [20]	Strong et al. [22]
Excessive alcohol consumption	Ingesting five or more doses (approximately 60 g alcohol) in the same time regardless of frequency.	YRBS [20]	Rehm et al. [23]
Smoking	Have smoked one or more cigarettes in the last 30 days.	YRBS [20]	WHO [24]
Sedentary behaviour	Remain four daily hours or more at the computer, television or video games on weekdays and weekends.	Questionnaire	Moraes et al. [25]; Silva et al. [10]
Unhealthy diet	Not meeting recommendations of one or more of the following items below: Animal fat (consuming up to three servings daily), sugar, salt, candies and snack (avoid daily consumption).	Fantastic [20]	Rodriquez Anez et al. [21]

Physical activity was assessed according to the number of at least 60-minute sessions in the past seven days, considering any type of physical activity that increased the heart rate and breathing of adolescents. Adolescents who practiced physical activities less than five times weekly were considered physically inactive. This parameter was based on evidence demonstrating that it is necessary to practice 60 minutes of physical activity five days a week for health maintenance during adolescence [22]. Note that the WHO recommends 60 minutes of exercise per day for adolescents. The threshold for excessive alcohol consumption was a daily intake frequency of five drinks or more on a single occasion during the past 30 days. The intake of approximately 60 g of alcohol results in a blood alcohol concentration of 0.8 g/L or more. The following were considered a dose of alcohol: a can of beer, a glass of wine, and a shot of whiskey, cachaça, rum, vodka or other. Those who reported consuming this amount on one or more days were considered to have this risky behaviour because excessive alcohol consumption on one occasion is already deleterious to health [23].

To assess smoking, the daily frequency in which individuals smoked in the past 30 days was questioned. Regular tobacco use was defined as having smoked at least one day during the 30 days preceding the survey; therefore, those who smoked one day or more were considered smokers [24]. Unhealthy diet was defined as the overconsumption of sugar, salt, animal fat, candies and snacks [21]. Excessive intake of animal fat was defined as the ingestion of more than three servings of meats and similar foods per day. The fact that sugar, salt, sweets and salty snacks are not recommended in balanced diets (consisting of grain and cereals, fruits and vegetables, meat, milk and dairy products) suggests that these foods are not considered healthy and should be avoided [21]. Individuals who consumed one or more of these items were considered to have an unhealthy diet.

Sedentary behaviour was assessed by the total screen time (sum of daily television, computer and video game time) on weekdays and the weekend. This variable was continuously collected and individuals who had four or more hours per day in front of the screen were considered to have this risky behaviour. This cut-off point for total screen time was used in other studies [10, 24, 25].

### Independent variables

A self-administered questionnaire, including questions of demographic (gender, age and skin colour) and economic variables (school shift and economic level), was applied. Sex (male and female), study shift (day and night) and skin colour (white, brown, black, yellow and indigenous) were self-reported. The skin colour categories were divided in accordance with the recommendations of the Brazilian Institute of Geography and Statistics [26]. However, for data analysis, skin colour was dichotomized into "White" (individuals who classified themselves as white) and “Others” (individuals who classified themselves as brown, black, yellow and indigenous were grouped into this single category) [27].

Age was continuously collected and dichotomized into 14–16 years old and 17–19 years old to ensure a homogeneous distribution of frequency in each category. The economic status was assessed by the purchasing power of families and classified into five categories: "A" (≥ $44,148.00), "B" (≥ $18,248.00 and < $44,148.00), "C" (≥ $6,284.00 and < $18,248.00), "D" and "E" (≥ $3,580.00 and < $6,284.00) [28]. For the present study, categories A and B were defined as "high" and the others as "low" [28].

### Statistical analysis

The heterogeneity chi-square test was used to evaluate differences between the groups for each lifestyle risk factor (Table 2 and S1 Dataset). The combinations of risk factors have been shown and the ratio between the observed and expected prevalence (O / E) was calculated [29]. The observed prevalence was identified for the sample of this study, and the expected prevalence was calculated by multiplying the individual probability of each risk factor based on its occurrence in the study population. Thus, it is possible to identify combinations that are above or below expectations [29] (Table 3).

**Table 2:** Risk factor characteristics of the adolescents according to demographic and socioeconomic variables.

**Variables**	**Full Sample**		**Physical inactivity**		**Excessive alcohol consumption**		**Smoking**		**Sedentary behaviour**		**Unhealthy diet**	
	n	(%)	n	(%)	n	(%)	n	(%)	n	(%)	n	(%)
**Total**	916		708	(77.3)	309	(33.7)	73	(7.9)	801	(87.4)	844	(92.1)
**Sex**				*p* = 0.001^a^		*p* = 0.959^a^		*p* = 0.392^a^		*p* = 0.001^a^		*p* = 0.007^a^
Male	408	(44.5)	295	(72.3)	138	(33.8)	36	(8.8)	379	(92.8)	365	(89.4)
Female	508	(55.4)	413	(81.3)	171	(33.6)	37	(7.2)	422	(83.0)	479	(94.2)
**Age (years)**				*p* = 0.256^a^		*p<*0.001^a^		*p* = 0.654^a^		*p* = 0.153^a^		*p* = 0.689^a^
14–16	542	(59.2)	426	(78.6)	155	(28.6)	45	(8.3)	481	(88.7)	501	(92.4)
17–19	374	(40.8)	282	(75.4)	154	(41.1)	28	(7.4)	320	(85.5)	343	(91.7)
**Skin colour**				*p* = 0.653^a^		*p* = 0.386^a^		*p* = 0.439^a^		*p* = 0.345^a^		*p* = 0.095^a^
White	578	(63.1)	444	(76.8)	189	(32.7)	43	(7.4)	510	(88.2)	526	(91.0)
Others	338	(36.9)	264	(78.1)	120	(35.5)	30	(8.8)	291	(86.0)	318	(94.0)
**School shift**				*p* = 0.066^a^		*p<*0.001^a^		*p* = 0.954^a^		*p* = 0.001^a^		*p* = 0.258^a^
Daytime	675	(73.7)	532	(78.8)	202	(29.9)	54	(8.0)	823	(89.7)	626	(92.7)
Night shift	241	(26.3)	176	(73.0)	107	(44.4)	19	(7.8)	300	(80.9)	218	(90.4)
**Economic Level**				*p* = 0.109^a^		*p* = 0.041^a^		*p* = 0.950^a^		*p* = 0.607^a^		*p* = 0.357^a^
High	637	(69.5)	483	(75.2)	229	(35.9)	51	(8.0)	561	(88.0)	590	(92.6)
Low	279	(30.5)	225	(80.6)	80	(28.6)	22	(7.8)	240	(86.0)	254	(91.0)

^a^ Heterogeneity chi-square test.

**Table 3:** Prevalence of combinations of health risk behaviors in the adolescent population.

**Risk Factors**	**Physical inactivity**	**Excessive alcohol consumption**	**Smoking**	**Sedentary behaviour**	**Unhealthy diet**	**n**	**Prevalence**		
							**Observed % (95%CI)**	**Expected % (95%CI)**	**O/E (95%CI)**
5	**+**	**+**	**+**	**+**	**+**	28	3.1 (1.9–4.1)	1.7 (0.8–2.4)	1.8 (1.1–2.6)
4	**-**	**+**	**+**	**+**	**+**	14	1.5 (0.7–2.3)	0.5 (0.1–0.9)	3.1 (2.0–4.2)
	**+**	**-**	**+**	**+**	**+**	11	1.2 (0.4–1.9)	3.3 (2.1–4.4)	0.4 (0.1–0.7)
	**+**	**+**	**-**	**+**	**+**	163	17.8 (15.3–20.2)	19.3 (16.7–21.8)	0.9 (0.3–1.5)
	**+**	**+**	**+**	**-**	**+**	08	0.9 (0.2–1.4)	0.2 (0.1–0.5)	3.6 (2.4–4.8)
	**+**	**+**	**+**	**+**	**-**	02	0.2 (0.1–0.5)	0.1 (0.1–0.3)	1.5 (0.7–2.3)
3	**-**	**-**	**+**	**+**	**+**	04	0.4 (0.1–0.8)	1.0 (0.3–1.5)	0.5 (0.1–0.8)
	**-**	**+**	**-**	**+**	**+**	46	5.0 (3.6–6.4)	5.7 (4.1–7.1)	0.9 (0.3–1.4)
	**-**	**+**	**+**	**-**	**+**	00	0.0 (0.0–0.0)	0.1 (0.1–0.2)	0.0 (0.0–0.0)
	**-**	**+**	**+**	**+**	**-**	01	0.1 (0.1–0.3)	0.1 (0.1–0.2)	2.6 (1.6–3.6)
	**+**	**-**	**-**	**+**	**+**	372	40.6 (37.4–43.7)	38.0 (34.8–41.1)	1.1 (0.4–1.7)
	**+**	**-**	**+**	**-**	**+**	02	0.2 (0.1–0.5)	0.5 (0.1–0.9)	0.5 (0.1–0.8)
	**+**	**-**	**+**	**+**	**-**	01	0.1 (0.1–0.3)	0.3 (0.1–0.6)	0.4 (0.1–0.7)
	**+**	**+**	**-**	**-**	**+**	21	2.3 (1.3–3.2)	2.8 (1.7–3.8)	0.8 (0.2–1.3)
	**+**	**+**	**-**	**+**	**-**	07	0.8 (0.2–1.3)	1.7 (0.8–2.4)	0.5 (0.1–0.8)
	**+**	**+**	**+**	**-**	**-**	00	0.0 (0.0–0.0)	0.1 (0.1–0.1)	0.0 (0.0–0.0)
2	**-**	**-**	**-**	**+**	**+**	99	10.8 (8.8–12.8)	11.2 (9.1–13.2)	1.0 (0.3–1.5)
	**-**	**-**	**+**	**-**	**+**	01	0.1 (0.1–0.3)	0.1 (0.1–0.3)	0.8 (0.2–1.3)
	**-**	**-**	**+**	**+**	**-**	00	0.0 (0.0–0.0)	0.1 (0.1–0.2)	0.0 (0.0–0.0)
	**-**	**+**	**-**	**-**	**+**	10	1.1 (0.4–1.7)	0.8 (0.2–1.4)	1.3 (0.6–2.0)
	**-**	**+**	**-**	**+**	**-**	04	0.4 (0.1–0.8)	0.5 (0.1–0.9)	0.9 (0.3–1.4)
	**-**	**+**	**+**	**-**	**-**	00	0.0 (0.0–0.0)	0.1 (0.1–0.1)	0.0 (0.0–0.0)
	**+**	**-**	**-**	**-**	**+**	56	6.1 (4.5–7.6)	5.5 (4.0–6.9)	1.1 (0.4–1.7)
	**+**	**-**	**-**	**+**	**-**	32	3.5 (2.3–4.6)	3.3 (2.1–4.4)	1.1 (0.4–1.7)
	**+**	**-**	**+**	**-**	**-**	00	0.0 (0.0–0.0)	0.1 (0.1–0.2)	0.0 (0.0–0.0)
	**+**	**+**	**-**	**-**	**-**	01	0.1 (0.1–0.3)	0.2 (0.1–0.5)	0.5 (0.1–0.8)
1	**-**	**-**	**-**	**-**	**+**	09	1.0 (0.3–1.6)	1.6 (0.7–2.4)	0.6 (0.1–1.0)
	**-**	**-**	**-**	**+**	**-**	16	1.8 (0.9–2.6)	1.0 (0.3–1.5)	1.8 (1.0–2.6)
	**-**	**-**	**+**	**-**	**-**	00	0.0 (0.0–0.0)	0.1 (0.1–0.1)	0.0 (0.0–0.0)
	**-**	**+**	**-**	**-**	**-**	01	0.1 (0.1–0.3)	0.1 (0.1–0.2)	1.6 (0.8–2.3)
	**+**	**-**	**-**	**-**	**-**	05	0.6 (0.1–1.0)	0.5 (0.1–0.9)	1.2 (0.5–1.8)
0	**-**	**-**	**-**	**-**	**-**	02	0.2 (0.08–0.5)	0.1 (0.1–0.3)	1.6 (0.8–2.3)

CI: Confidence interval; + presence of risk behavior;–absence of risk behavior; O: Observed prevalence; E: Expected prevalence; O/E: Ratio between observed and expected prevalence.

Associations between the dependent variable "clustering of risk factors" and both demographic and economic variables were analysed by multinomial logistic regression, with estimations of the odds ratio (OR) and respective confidence intervals (CI 95%). The reference category was "no risk factor"; however, due to the low frequency of individuals in this category (0.2%), it was combined with the category "one risk factor" (3.7%). Therefore, "none or one risk factor" was considered the reference (3.9%). The adjusted analysis controlled for all independent variables of the study (sex, age, skin colour, school shift and economic level) (Table 4). Moreover, binary logistic regression was performed for simultaneous risk factors that had a higher prevalence in this population (6.0% of adolescents had both physical inactivity and unhealthy diet, 10.8% of adolescents had sedentary lifestyle and unhealthy diet, 40.5% had physical inactivity, sedentary behaviour and unhealthy diet, and 17.7% had physical inactivity, excessive alcohol consumption, sedentary behaviour and unhealthy diet), along with the estimated OR and respective 95% confidence interval. The adjusted analysis controlled for all independent variables of the study (sex, age, skin colour, school shift and economic level) (Table 5). We adopted p < 0.05 as the level of significance for all statistical tests, and we used Stata®13.0 (College Station, Texas, USA) and SPSS® 21.0 (Armonk, New York, USA).

**Table 4:** Association between risk factors and demographic and socioeconomic variables in adolescents.

	Two risk factors^a^		Three risk factors^a^		Four risk factors^a^		Five risk factors^a^	
	n (%)	OR (95%CI)^b^	n (%)	OR (95%CI) ^b^	n (%)	OR (95%CI) ^b^	n (%)	OR (95%CI) ^b^
**Total**	204 (22.2)		452 (49.3)		199 (21.7)		29 (3.1)	
**Sex**								
Male	96 (23.5)	1.00	193 (47.3)	1.00	88 (21.5)	1.00	15 (3.6)	1.00
Female	108 (21.2)	1.10 (0.52–2.34)	259 (50.9)	1.31 (0.63–2.70)	111 (21.8)	1.28 (0.60–2.71)	14 (2.7)	0.93 (0.34–2.57)
**Age (years)**								
14–16	121 (22.3)	1.00	277 (51.1)	1.00	108 (19.9)	1.00	17 (3.1)	1.00
17–19	83 (22.1)	1.04 (0.47–2.27)	175 (46.7)	0.98 (0.46–2.10)	91 (24.3)	1.32 (0.60–2.91)	12 (3.2)	1.06 (0.37–3.06)
**Skin colour**								
White	136 (23.5)	1.00	277 (47.9)	1.00	126 (21.8)	1.00	17 (2.9)	1.00
Others	68 (20.1)	1.08 (0.48–2.42)	175 (51.7)	1.37 (0.63–2.96)	73 (21.6)	1.25 (0.56–2.80)	12 (3.5)	1.55 (0.54–4.45)
**School shift**								
Daytime	148 (21.9)	1.00	338 (50.0)	1.00	146 (21.6)	1.00	21 (3.1)	1.00
Night shift	56 (23.2)	0.81 (0.35–1.87)	114 (47.3)	0.74 (0.33–1.65)	53 (21.9)	0.75 (0.32–1.74)	08 (3.3)	0.84 (0.27–2.66)
**Economic Level**								
High	138 (21.6)	1.00	309 (48.5)	1.00	145 (22.7)	1.00	22 (3.4)	1.00
Low	66 (23.6)	1.22 (0.53–2.82)	143 (51.2)	1.18 (0.52–2.63)	54 (19.3)	0.93 (0.40–2.16)	07 (2.5)	0.81 (0.25–2.58)

OR: Odds ratio; CI: Confidence interval

^a^Reference category: zero and one risk factor (n = 32).

^b^Adjusted analysis for all independent variables.

**Table 5:** Association between combinations of health risk behaviors most prevalent and demographic and socioeconomic variables in adolescents.

	**PI + UD**			**SB + UD**			**PI + SB + UD**			**PI + EAC + SB + UD**		
	n (%)	OR (95%CI)^a^	p	n (%)	OR (95%CI)^a^	p	n (%)	OR (95%CI)^a^	p	n (%)	OR (95%CI)^a^	p
**Total**	55 (6.0)			99 (10.8)			371 (40.5)			163 (17.7)		
**Sex**			**<0.01**			**0.01**			0.45			0.38
Male	12 (2.9)	**1.00**		56 (13.7)	**1.00**		157 (38.4)	1.00		69 (16.9)	1.00	
Female	43 (8.4)	**3.03 (1.57–5.85)**		43 (8.4)	**0.58 (0.38–0.89)**		214 (42.1)	1.10 (0.84–1.45)		94 (18.5)	1.16 (0.82–1.64)	
**Age (years)**			0.27			0.80			**<0.01**			**0.02**
14–16	29 (5.3)	1.00		60 (11.7)	1.00		246 (45.3)	**1.00**		84 (15.5)	**1.00**	
17–19	26 (6.9)	1.37 (0.77–2.42)		39 (10.4)	0.94 (0.60–1.46)		125 (33.4)	**0.66 (0.49–0.87)**		79 (21.1)	**1.49 (1.05–2.12)**	
**Skin colour**			0.31			0.10			0.70			0.82
White	31 (5.3)	1.00		70 (12.1)	1.00		231 (39.7)	1.00		104 (17.9)	1.00	
Others	24 (7.1)	1.33 (0.76–2.32)		29 (8.5)	0.68 (0.43–1.08)		140 (41.4)	1.05 (0.80–1.39)		59 (17.4)	0.96 (0.67–1.36)	
**School shift**			0.91			0.48			**<0.01**			0.98
Daytime	39 (5.7)	1.00		76 (11.2)	1.00		298 (44.1)	**1.00**		118 (17.4)	1.00	
Night shift	16 (6.6)	1.03 (0.55–1.94)		23 (9.5)	0.83 (0.50–1.38)		73 (30.2)	**0.59 (0.43–0.82)**		45 (18.6)	1.00 (0.67–1.48)	
**Economic Level**			**0.03**			0.50			0.19			0.21
High	30 (4.7)	**1.00**		73 (11.4)	1.00		252 (39.5)	1.00		119 (18.6)	1.00	
Low	25 (8.9)	**1.83 (1.05–3.21)**		26 (9.3)	0.84 (0.52–1.36)		119 (42.6)	1.21 (0.90–1.62)		44 (15.7)	0.78 (0.53–1.15)	

PI: Physical inactivity; UD: Unhealthy diet; SB: Sedentary behaviour; EAC: Excessive alcohol consumption; OR, Odds Ratio; CI, Confidence Interval.

^a^Adjusted analysis for all independent variables.

## Results

Overall, 916 adolescents aged 16.1 ± 1.1 years were analysed. Most subjects were female (55.4%), aged 14–16 years (59.2%), of white skin colour (63.1%), students of the day shift (73.7%) and of a high economic level (69.5%). About eight in ten adolescents did not perform physical activity on a regular basis (77.3%) and had sedentary behaviour equal to or greater than four hours (87.4%). One third of adolescents used alcohol excessively (33.7%), and one in 10 adolescents smoked cigarettes (7.9%). The consumption of unhealthy food was observed in 92.1% of adolescents (Table 2).

Female adolescents had a higher prevalence of physical inactivity (81.3%) than male adolescents (72.3%). Older individuals, those of a skin colour other than white, those of the night shift and those of a high economic level had a higher alcohol consumption than younger individuals, those of white skin colour, those of the day shift and those of a lower economic level. Boys and students of the day shift had a higher prevalence of sedentary behaviour (92.8% and 89.7%, respectively) than girls and students of the night shift (83.0% and 80.9%, respectively). Girls demonstrated a higher prevalence of unhealthy diet (94.2%) than boys (89.4%) (Table 2).

The simultaneous prevalence of five risk factors (3.1%; CI = 1.9%-4.1%) was approximately twice as high as expected (1.7%; CI = 0.8%-2.4%). By analysing the clustering of four risk factors, we found a prevalence three times higher than expected when excessive alcohol consumption, smoking, sedentary behaviour and unhealthy diet were combined (observed prevalence = 1.5%, CI = 0.7%-2.3%; and expected prevalence = 0.5%, CI = 0.1%-0.9%) and also when physical inactivity, excessive alcohol consumption, smoking and unhealthy diet were combined (observed prevalence = 0.9%, CI = 0.2%1.4%; and expected prevalence = 0.2%, CI = 0.1%-0.5%). For three simultaneous risk behaviours, i.e., excessive alcohol consumption, smoking and sedentary behaviour, the prevalence was three times higher than expected (O/E ratio = 2.6%; CI = 1.6%-3.6%). For two simultaneous behaviours, the prevalence of excessive alcohol consumption and unhealthy diet combined had a high O/E ratio (1.3%; CI = 0.6%-2.0%) (Table 3).

The prevalences of adolescents with no, one, two, three, four, and five risk factors were 0.2%, 3.7%, 22.2%, 48.5%, 22.1% and 3.1%, respectively. In the comparison of demographic and economic characteristics of adolescents who had two, three, four or five risk factors with those who had no or one risk factor, no significant differences were found (Table 4).

Adolescents had a higher prevalence of the following behaviour patterns: physical inactivity and unhealthy diet (5.8%); sedentary behaviour and unhealthy diet (10.7%); physical inactivity, excessive alcohol consumption, unhealthy diet and sedentary behaviour (18.4%); and physical inactivity, unhealthy diet and sedentary behaviour (40.1%) (Table 5). The simultaneous presence of physical inactivity and unhealthy diet was three times higher among girls (OR = 3.03; CI = 1.57–5.85) than that among boys and was almost twice as high among low income individuals (OR = 1.83; CI = 1.05–3.21) than that among high income individuals. Girls were also less likely to have sedentary behaviour and concomitant unhealthy diet (OR = 0.58; CI = 0.38–0.89) than boys. Older individuals and students of the night shift were less likely (OR = 0.66; CI = 0.49–0.87 and OR = 0.59; CI = 0.43–0.82, respectively) to present the combination of physical inactivity, unhealthy diet and sedentary behaviour than their respective counterparts. Older adolescents were more likely to have four risk factors (physical inactivity, excessive alcohol consumption, unhealthy diet and sedentary behaviour; OR = 1.49; CI = 1.05–2.12).

## Discussion

Approximately nine out of ten adolescents had two or more risk factors for NCDs in this study. These results encourage the policies and recommendations of the WHO and Ministry of Health of Brazil regarding the importance of performing regular physical activity, reducing sedentary behaviour and eating healthily in all age groups [6, 14].

Among the risky behaviours analysed in the present study, the four most prevalent combinations included the behaviours of physical inactivity, sedentary lifestyle, unhealthy diet and excessive alcohol consumption. These behaviours are classified among the main risk factors for morbidity and mortality in adults, which is worrying because the behaviours adopted during adolescence tend to remain in adulthood [6].

Girls were more likely to have the simultaneous behaviours of physical inactivity and unhealthy diet than boys. This finding was also reported in another study conducted in Brazil [30]. Evaluating physical activity and nutrition is a complex task. For example, to determine whether nutrition is appropriate, different indicators of diet may be evaluated, such as the frequency of fruit and vegetable consumption [5], the frequency of fibre intake [30] or the consumption of beans and legumes [6]. To evaluate physical activity, the methods can be evaluated directly or indirectly, they may vary according to the daily periods of moderate or vigorous physical activity [11], or they can specifically include activities in different domains, such as physical activity during leisure time and during the commute to school [30]. Therefore, the generalization of our results and the comparison with those obtained from other studies should be performed with caution because the methods used to evaluate these health behaviours are very specific.

Girls in this study were also less likely to have simultaneous sedentary behaviour and unhealthy diet than boys. A study of 1,484 adolescents aged 13–16 years in the UK identified that boys spent more time with televisions, computers and video games, while girls spent more time studying [31]. The literature shows that the excessive time that the boys spent in front of such electronic devices promotes the consumption of sweets, snacks and soft drinks [32]. This fact justifies the results of our study.

Adolescents of low income were more likely to present the simultaneous behaviours of physical inactivity and unhealthy diet. Individuals with low income are less likely to attend environments with adequate infrastructure for physical activity and sports, have less access to supervised exercise programmes and have lower purchasing power to buy sporting goods [33]. Individuals of lower socioeconomic status also tend to have less education and less access to information (such as knowledge of the benefits of physical activity and healthy eating) and, thus, tend to have less healthy habits [33, 34]. In addition, financial resources are related to the nutritional quality of diets, as maintaining an appropriate diet is costly and is therefore less frequent among population groups with lower income [34].

Older individuals were less likely to have simultaneous physical inactivity, unhealthy diet and sedentary behaviour. Studies examining the relationship between simultaneous risk factors and age reported that older adolescents tended to have more risk behaviours; however, the probability of this specific combination (i.e., physical inactivity, sedentary behaviour and unhealthy diet) was analysed only in one study [12]. The literature shows that older individuals showed a higher prevalence of these behaviours individually [35]; however, when analysing the occurrence of these behaviours simultaneously in this study, contradictory results were found. These differences may arise from the possibility that factors associated with isolated behaviours are not the same when the behaviours analysed in association.

Older individuals were more likely to have simultaneous physical inactivity, excessive alcohol consumption, unhealthy diet and sedentary behaviour. Some studies have found that older adolescents tend to have a higher prevalence of risk behaviours as the amount of associated factors increases [11, 35]. These results are due to the exposure to stressful situations and social pressures during the late stage of adolescence, where individuals become more independent in their choices, accumulate more responsibility, and live in new environments [36].

Adolescents of the night shift were less likely to have simultaneous physical inactivity, sedentary behaviour and unhealthy diet than students of the day shift. Individuals studying at night usually worked during the day [37]. Thus, it is assumed that students of the night shift have higher energy expenditure, arising from work activities and even from the commute to work.

Although studies do not usually report the combination of physical inactivity, excessive alcohol consumption, unhealthy diet and sedentary behaviour, inferences could be made to understand the relationship between these behaviours because it was one of the most common combinations among adolescents of this study. Young people consume alcohol due to peer pressure, when socializing, for anxiety, depression and stress reduction and in search of its effects (i.e., relaxation, drunkenness and loss of inhibition) [38]. The practice of physical activity provides psychological and social benefits; therefore, inactive individuals do not benefit from these aspects and seek other ways to meet these needs, for example, by consuming alcohol. Moreover, the consumption of alcohol is culturally accompanied by inadequate nutrition [39]. Finally, individuals who spend more time in front of the screen are more exposed to pressure from the media through images of pleasure and sociability among drinkers [40].

Among the study limitations, behaviours were assessed by indirect measures (i.e., self-reported questionnaire). Although this method is commonly used in epidemiological studies due to its easy application and low cost, indirect measures depend on memory, interpretation and the willingness of individuals [41]. Besides, the questionnaires used in this study were not reflecting the chronic conditions (such smoking, physical activity and alcohol consumption). Thus, the results may be biased, requiring caution when extrapolating data for different contexts [41]. Another limitation was small sample size for multiple comparisons between the risk factors. Thus, the results should be considered with caution.

Most of the previous studies that evaluated the clustering of risk factors in adolescents did not include the variable of sedentary behaviour. The interest in this behaviour is recent, but evidence has shown its effect on adolescent health [14]; therefore, this study contributes to the understanding of sedentary behaviour in conjunction with other risk factors. In addition, the sample representativeness ensures the identification of groups with these lifestyle risk factors among young people of this region, which guides the planning of interventions aimed at the reduction of more than one risk factor.

It could be concluded that adolescents had a high prevalence of simultaneous risk factors for NCDs; 70.7% of students had two or three factors, and 24.8% had four or five risk factors. Among the most prevalent simultaneous behaviours, unhealthy diet and physical inactivity were found to be higher among girls and poorer individuals, while unhealthy diet and sedentary behaviour were found to be higher among boys. Moreover, younger adolescents and those of the day shift were more likely to be simultaneously physically inactive, sedentary and consumers of unhealthy foods. Older adolescents (17–19 years old) were more likely to present simultaneously behaviours of physical inactivity, excessive alcohol consumption, unhealthy diet and sedentary behaviour.

## Supporting Information

S1 Dataset (XLSX)
